# Epicardial adipose tissue is a robust measure of increased risk of myocardial infarction – a meta-analysis on over 6600 patients and rationale for the EPIC-ACS study

**DOI:** 10.1097/MD.0000000000028060

**Published:** 2021-12-30

**Authors:** Stefanie Hendricks, Iryna Dykun, Bastian Balcer, Matthias Totzeck, Tienush Rassaf, Amir Abbas Mahabadi

**Affiliations:** West German Heart and Vascular Center Essen, Department of Cardiology and Vascular Medicine, University Hospital Essen, Essen, Germany.

**Keywords:** computed tomography, coronary events, echocardiography, epicardial adipose tissue, myocardial infarction, pericardial adipose tissue

## Abstract

**Background:**

Epicardial adipose tissue (EAT) surrounds the heart and the coronary vessels. EAT produces pro- and anti-inflammatory cytokines. Several studies have already documented the association of EAT and cardiovascular risk factors as well as coronary artery disease manifestations. Currently computed tomography (CT) is considered the gold standard for measurement of 3-dimensional volume of EAT. In addition, echocardiography might be an easy accessible alternative in particular in an emergency setting.

**Methods:**

We performed a metaanalysis of existing studies describing the differences of EAT in patients with and without myocardial infarction. We used established databases and were searching for “epicardial adipose tissue” or “pericardial adipose tissue” and “myocardial infarction”, “coronary events”, or “acute coronary syndrome”. We included over 6600 patients from 7 studies. Random effect models were calculated and all analyses were performed by using the Review Manager 5.3.

**Results:**

Patients with myocardial infarction had 37% (confidence interval [0.21-0.54], *P* value <.001)] higher measures of EAT compared to patients without myocardial infarction. Comparing studies using echocardiography vs CT for assessment of EAT thickness, similar relative differences in EAT with wide overlap of confidence intervals were observed (for echocardiography: 0.4 [0.04-0.76], for CT: 0.36 [0.16-0.57], *P* value <.001 for both).

**Conclusions:**

Patients with myocardial infarction have more EAT as compared to patients without myocardial infarction independently of the used imaging modality. Further prospective studies are needed to evaluate, how quantification of EAT in clinical routine can improve patients management.

## 1 Introduction

Epicardial adipose tissue (EAT) surrounds the heart and the coronary vessels. It is metabolically active, secreting pro- and anti-inflammatory mediators and cytokines.[^1^,^2^,^3^] With increasing amount of EAT, the balance between pro- and anti-inflammatory markers shifts toward a more pro-inflammatory state.^[4]^ As such, EAT is suggested to locally influence atherosclerosis development. Available data could demonstrate that EAT is associated with cardiovascular risk factors, measures of subclinical atherosclerosis and prevalent as well as incident coronary artery disease manifestation.^[5]^

Currently, computed tomography (CT) is considered the gold standard for the assessment EAT, allowing for quantification of its 3-dimensional volume.[^6^,^7^] In addition, echocardiography based EAT thickness is an easy accessible alternative in particular in an emergency setting. While studies using both CT and echocardiography have been published in various cohorts over the last decade, currently no comparison of CT-based EAT volume and echocardiography based EAT thickness exists. In addition, despite the overwhelming evidence, documenting the strong association of EAT with acute coronary syndromes, no study has aimed to evaluate, whether assessment of EAT may alter clinical decision-making. Therefore, currently quantification EAT is not implemented into clinical routine of patients presenting with acute chest pain.

We therefore performed a meta-analysis on existing studies, comparing EAT in patients with and without myocardial infarction. Specifically, we aimed to compare effect sizes from CT-derived and echo-derived quantification of EAT. In addition we describe the rationale for the “Epicardial adipose tissue thickness PredIcts obstructive Coronary artery disease in Acute Coronary Syndrome patients (EPIC-ACS) study”, a prospective observational study to investigate the impact of EAT quantification by echocardiography to predict significant coronary artery disease in patients presenting with acute chest pain.

## 2 Methods

This study was performed following the Preferred Reporting Items for Systematic Reviews and Meta-Analyses (PRISMA) guidelines^[8]^ and in accordance with the “Meta-analysis Of Observational Studies in Epidemiology (MOOSE)” recommendations,^[9]^ and the Cochrane Handbook for Systematic Reviews of Interventions.^[10]^ The study was registered with PROSPERO (international prospective register of systematic reviews; CRD42018114433).

### 2.1 Data sources and searches

We performed a systematic search using the Pubmed, Cochrane, SCOPUS, and Web of Science databases for studies, describing EAT in patients with and without myocardial infarction. Manuscripts, published until October 1, 2018, were included. We made our search specific and sensitive using Medical Subject Headings terms and free text and considered studies published in English language. Search terms used were “epicardial adipose tissue”, “pericardial adipose tissue”, “myocardial infarction”, “coronary events”, and “acute coronary syndrome”.

### 2.2 Data selection

We included studies reporting specific values for EAT measurements in patients with and without myocardial infarction as well as the corresponding number of patients. Information on imaging technique for EAT measurement including echocardiography, computed tomography or magnetic resonance imaging was collected. There were no restrictions to comorbidities. Two authors (SH and AM) independently reviewed the titles and abstracts of the studies, followed by full text screening to identify the studies meeting inclusion criteria. The study collection was supervised by TR and a consensus was negotiated in case of disagreement.

### 2.3 Data extraction

Data extraction was performed by SH and AM. A prespecified form was used for the data extraction. The following data were collected: year of publication, overall sample size, mean age, percent male, type of imaging modality, study design (prospective vs cross-sectional), number of patients with and without myocardial infarction, EAT for patients with and without myocardial infarction.

### 2.4 Data analysis

Mean age and percent male was calculated for the sum of participants from each study. EAT measure in patients with and without myocardial infarction was compared. For comparability of different EAT measures, EAT measures were normalized to mean values for patients without myocardial infarction for each study separately. Subgroup analyses were performed, stratifying by imaging modality used and study design (prospective vs cross-sectional). Random effect models were calculated. As it is not suggested to perform meta-regression if fewer than 10 studies are included in a meta-analysis^[10]^ we decided to forego an additional meta-regression. All analyses were performed using Review Manager 5.3 (The Cochrane Collaboration).

## 3 Results

**Figure 1:**
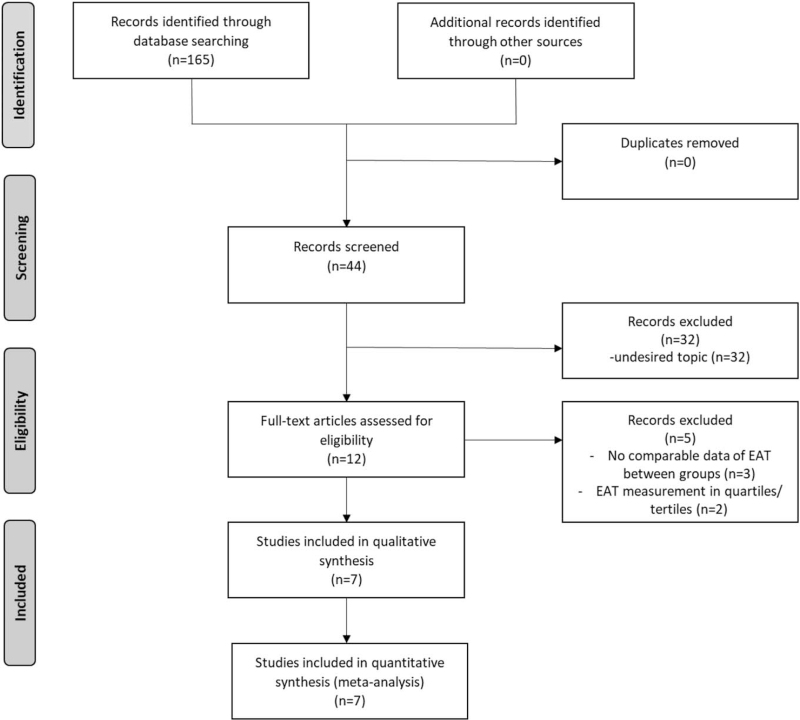
Flow-chart for the search strategy of selected studies. EAT = epicardial adipose tissue.

The initial electronic search resulted in a total of 165 hits. After removing duplicates, 84 articles were evaluated. After exclusion of review articles, case series, irrelevant citations, and publications not in English language, 12 manuscripts were evaluated as full-texts. Of those, 3 studies were excluded as EAT measures were not reported comparing patients with and without myocardial infarction and additional 2 studies were excluded for reporting EAT measurements in quartiles/tertiles only. The flow-chart for the search strategy of selected studies can be found in Figure 1.

**Figure 2:**
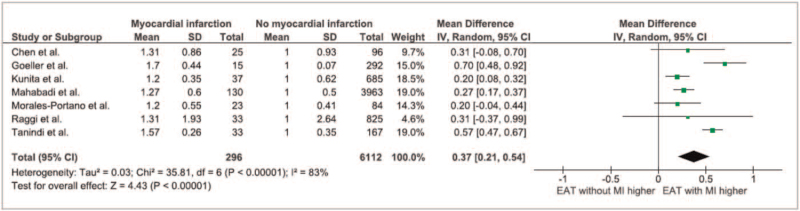
Forest plot of all included studies. EAT = epicardial adipose tissue.

Overall, 6641 patients (mean aged 58.9 years, 53% male) from 7 studies were included (Table 1).[^11^,^12^,^13^,^14^,^15^,^16^,^17^] Of those, 296 patients had either a myocardial infarction at inclusion for cross-sectional studies (n = 128 events) or developed a myocardial infarction during follow-up for longitudinal studies (n = 168 events). Patients with myocardial infarction had 37% higher measures of EAT compared to patients without myocardial infarction (95% confidence interval: 21% to 54%, Fig. 2). Studies on CT added 5980 participants from 4 studies[^12^,^13^,^14^,^16^] to the analysis (215 myocardial infarctions), whereas only 251 patients from 2 studies using echocardiography[^15^,^17^] were included (56 myocardial infarctions). This imbalance between CT and echocardiography based studies was predominantly caused by the study by Mahabadi et al,^[14]^ including 4093 subjects. The differences in studies led to a significant heterogeneity of the analysis (*I* ^2^ = 83%).

**Table 1:** Key study characteristics of included studies.

First author	N of patients (age in yrs, male n,%)	N of patients with myocardial infarction	Imaging method for EAT quantification	Study design	Study cohort	Ref.
Chen et al 2015	220; 64.5 + 13.3; 125 (56.8)	25	MRI	Retrospective	Consecutive patients who underwent MRI and coronary angiography within 12 mo	^[9]^
Goeller et al 2018	317; 60.3 ± 8.3; 291 (64)	15	CT	Prospective	Randomly selected subjects from the single-center EISNER trial (n = 2614)	^[10]^
Kunita et al 2014	732; 65.0 ± 10.9 442 (61)	37	CT	Retrospective	Patients without proven coronary artery disease who underwent non-contrast cardiac CT	
Mahabadi et al 2013	4093; 59.4; 1928 (47.0)	130	CT	Prospective	Based on the population-based prospective cohort Heinz Nixdorf Recall study	^[11]^
Morales-Portano et al 2018	107; 63.6 ± 9.67, 86 (80.4)	23	Echo	Prospective	Observational, longitudinal, single-center study	^[12]^
Raggi et al 2015	843; 50 ± 8, 581.7 (69)	33	CT	Retrospective	Observational study of consecutive HIV-infected patients receiving antiretroviral therapy for at least 6 mo	^[13]^
Tanindi et al 2015	200; 59.83; 96 (48.68)	33	Echo	Retrospective	Consecutive patients with stable angina pectoris or acute coronary syndrome	^[14]^

**Figure 3:**
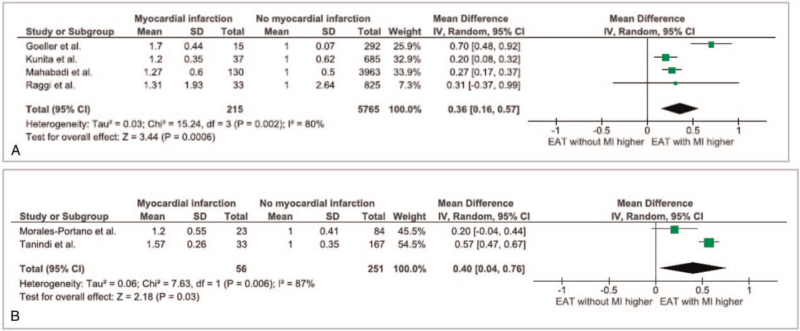
Forest plot for the association of EAT measures with myocardial infarction, stratified by imaging method (Echocardiography (A) vs CT (B)). CT = computed tomography, EAT = epicardial adipose tissue.

Comparing studies using echocardiography vs CT for assessment of EAT thickness, similar relative differences in EAT with wide overlap of confidence intervals were observed (Echocardiography measures: 40 [4%-76%], CT measures: 36 [16%-57%], Fig. 3).

**Figure 4:**
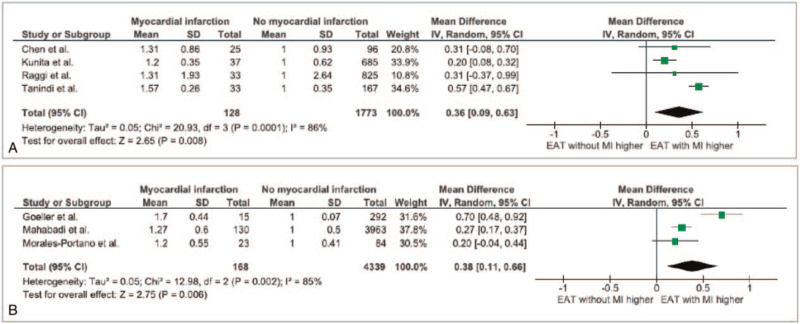
Forest plot for the association of EAT measures with myocardial infarction, stratified by study design (cross sectional (A) vs prospective (B)). EAT = epicardial adipose tissue.

In addition, we compared effect sizes from prospective[^12^,^14^,^15^] and cross-sectional studies.[^11^,^13^,^16^,^17^] We again observed comparable effect sizes from prospective and cross-sectional studies (38 [11-66] vs 36 [9-63], for prospective and cross-sectional studies, respectively, Fig. 4). We performed publication bias analysis, as depicted in Figure S1, Supplemental Digital Content and found that publication bias is not of concern. In sensitivity analysis, we removed the study by Mahabadi et al^[14]^ from the meta-analysis as this, due to its size, may have biased the results and observed comparable effect sizes (Figure S2, Supplemental Digital Content).

In Newcastle Ottawa Scale for quality assessment high quality was documented in the majority all included studies (Figure S3, Supplemental Digital Content). In GRADE approach all included studies were categorized in a low rating of quality due to their observational study design. However, no factors that would decrease the quality of evidence occurred, large and similar effect sizes were observed in all included studies, adding to the quality of evidence.

## 4 Rationale for the EPIC-ACS study

There is overwhelming evidence, documenting the strong association of EAT with acute coronary syndromes. However, no study has so far aimed to evaluate, whether assessment of EAT may alter clinical decision making. Therefore, currently EAT is not implemented into clinical routine of patients presenting with acute chest pain. The “Epicardial adipose tissue thickness PredIcts obstructive Coronary artery disease in Acute Coronary Syndrome patients (EPIC-ACS) study” is a prospective observational study, aiming to investigate, whether quantification of EAT thickness via transthoracic echocardiography enables improved risk stratification in patients presenting with acute chest pain to the emergency department. The study hypothesizes that quantification of EAT via a quick and easy echocardiography-based measurement improves prediction of a coronary cause of chest pain, ultimately suggesting altered patient management. The study hypothesizes that quantification of EAT via a quick and easy echocardiography based measurement improves prediction of a coronary cause of chest pain, ultimately suggesting altered patient management.

### 4.1 Methods of the EPIC-ACS study

The EPIC-ACS study prospectively includes consecutive patients, presenting to the emergency department of the University Hospital Essen with acute chest pain suggestive of an acute coronary syndrome. Exclusion criteria are known obstructive coronary artery disease prior to presentation, prior revascularization therapy, ST-elevation myocardial infarction, or unwillingness to provide informed consent. The primary endpoint is defined as need for coronary revascularization therapy (PCI/stent or bypass) within 30 days after presentation. A sample size of n = 653 participants is anticipated to achieve 80% power to detect an odds ratio of 1.3 for the primary endpoint for patients with EAT ≥ median (event rate 0.5) as compared to patients with EAT < median (event rate 0.385) with a drop-out rate of 10% (type-I error probability: 5%). EAT-thickness will be quantified using 2-dimensional transthoracic echocardiography, performed by standard echocardiography systems without the use of specific applications (Philips CX 50 or Philips Sparq system, Philips Healthcare, Best, the Netherlands). EAT is defined as space between the outer wall of the myocardium and the visceral layer of the pericardium. Measurement of EAT thickness perpendicular to the free wall of the right ventricle at end-systole in 2 cardiac cycles in parasternal long- and short-axis views. Mean and maximal EAT thickness will be used for further assessment. The study has been registered online (NCT03787797). The EPIC-ACS study was approved by the institutional ethics committee (18-8198-BO).

### 4.2 Analysis plan

We aim to determine the distribution of EAT thickness. Descriptive statistics of EAT and co-variables, stratified by presence of obstructive coronary artery disease (CAD) will be performed. Afterwards, univariate and multivariate linear regression analysis for the association of EAT thickness with presence of obstructive CAD as primary endpoint will be performed. In predefined exploratory analysis, assessment of the value of EAT thickness for prediction of obstructive CAD via receiver operating characteristics, adding EAT thickness over established risk scores is anticipated. Analysis of secondary endpoints will be performed using identical tests as for the primary endpoint.

## 5 Discussion

Within the meta-analysis, we could demonstrate that EAT is increased in patients with myocardial infarction, the effect sizes are comparable between echocardiography and CT for measurements of EAT. Our results suggest that echocardiography can be used as a quick and easy alternative for quantification of EAT instead of CT, enabling its assessment in an emergency setting. With the EPIC-ACS study, using echocardiography for quantification of EAT in patients with acute chest pain, we aim to evaluate, whether assessment of EAT in this cohort can lead to improved prediction of coronary causes of chest pain and may ultimately alter patient management.

Several studies have documented the association of EAT with coronary artery disease manifestation.[^5^,^18^,^19^,^20^] Higher measures of EAT can already be detected in obese children and adolescents,[^21^,^22^] newest data even demonstrated the development of EAT in embryos.^[23]^ EAT is associated with cardiovascular risk factors and was found to be associated with the TIMI risk score and Syntax II score but not GRACE score in the setting of acute coronary syndrome.^[24]^ In particular, computed tomography is used for the assessment of EAT volume. While several studies use echocardiography as the imaging modality of choice for the assessment of EAT thickness, to date, no data compared different imaging modalities for quantification of epicardial adipose tissue in patients with and without myocardial infarction. In the present meta-analysis, we demonstrate a significant difference in EAT in patients with and without myocardial infarction, independent of the used imaging technology, despite different study designs and included cohorts. Therefore, EAT measurement may gain in importance for risk stratification and will provide a therapeutic target in the future.^[25]^ In addition, further studies are needed to implement prevention strategies to modulate coronary inflammation.^[26]^

CT is currently considered as gold standard for quantification of EAT. The advantages of CT are a 3-dimensional measurement of EAT volume while echocardiography routinely assessed EAT only in a 2-dimensional way. In addition, CT can measure the density of EAT.^[27]^ On the other hand, CT is a more expensive and makes high demands on technical as well as personnel resources. In addition, it requires radiation exposure to the patient. In contrast, echocardiography is a quick and broadly available alternative in daily clinical routine. Furthermore, there is no radiation exposition in using echo. In our analysis, we observed that effect sizes were similar independently of the used imaging technology. Therefore, our data suggests that echocardiography could be used as an easy alternative especially in an emergency setting.

### 5.1 Implications

Independent of study design and imaging modality used, EAT is increased in patients with acute coronary syndrome. Our results suggest that echocardiography based assessment of EAT thickness as easy accessible imaging in emergency room settings, may qualify for risk assessment of patients with suspected acute coronary syndrome. Despite the overwhelming data, EAT is currently not implemented into clinical routine of patients presenting with acute chest pain. The prospective observational EPIC-ACS study, we will provide robust evidence, evaluating the ability of EAT quantification by echocardiography to assess the patient's pretest probability and ultimately evaluate the impact of EAT assessment on patient management. The results may help to therefore understand the clinical value of EAT quantification in the workup of patients presenting with acute chest pain in clinical routine.

### 5.2 Limitations

Overall, the meta-analysis is limited by number of studies included, especially when considering studies using echocardiography. Several studies on the topic of EAT and myocardial infarction were excluded, as key information was not provided. The heterogeneity of studies included, was of additional concern. Likewise, cohorts of included studies differentiated, in particular in form of primary preventive cohorts and symptomatic cohorts. Both studies using echocardiography as imaging modality included consecutive patients with stable or unstable angina.[^15^,^17^] Notably Raggi et al^[16]^ examined HIV-infected patients receiving antiretroviral therapy. However, we observed robust effect sizes, independent of imaging modality and study design. As a further limitation, comparison of imaging modality excluded patients using magnetic resonance imaging, as only a single study was available.^[11]^

### 5.3 Future directions

Prospectively, future studies should assess the value of routinely performed quantification of epicardial adipose tissue via echocardiography as an easily available imaging modality for risk stratification of patients presenting with suspected acute coronary syndrome. Therefore, we initiated the EPIC-ACS study to evaluate whether the assessment of epicardial adipose tissue may alter patient's management.

## 6 Conclusion

In the present meta-analysis, the data showed that EAT is increased in patients with myocardial infarction independent of the used imaging modality. The data suggests that quantification of EAT thickness using echocardiography is an easily accessible alternative in clinical settings compared to CT-derived 3-dimensional EAT volume. The EPIC-ACS study aims to investigate, whether quantification of EAT in the emergency setting of patients with acute chest pain improves prediction of coronary artery disease and may alter patient management.

## Author contributions

**Conceptualization:** Stefanie Hendricks, Amir Abbas Mahabadi.

**Data curation:** Stefanie Hendricks, Iryna Dykun.

**Formal analysis:** Iryna Dykun, Amir Abbas Mahabadi.

**Investigation:** Bastian Balcer.

**Methodology:** Stefanie Hendricks, Iryna Dykun, Bastian Balcer.

**Project administration:** Tienush Rassaf.

**Resources:** Tienush Rassaf.

**Supervision:** Tienush Rassaf, Amir Abbas Mahabadi.

**Validation:** Matthias Totzeck.

**Writing – original draft:** Stefanie Hendricks, Amir Abbas Mahabadi.

**Writing – review & editing:** Iryna Dykun, Bastian Balcer, Matthias Totzeck, Tienush Rassaf.

## Supplementary Material

Supplemental Digital Content

## Supplementary Material

Supplemental Digital Content

## Supplementary Material

Supplemental Digital Content
