# Supplementary figures and images for: Epicardial adipose tissue is a robust measure of increased risk of myocardial infarction – a meta-analysis on over 6600 patients and rationale for the EPIC-ACS study
Source: Medicine (Baltimore). 2021 Dec 30;100(52):e28060. doi: 10.1097/MD.0000000000028060 (PMC8718235; doi:10.1097/MD.0000000000028060)

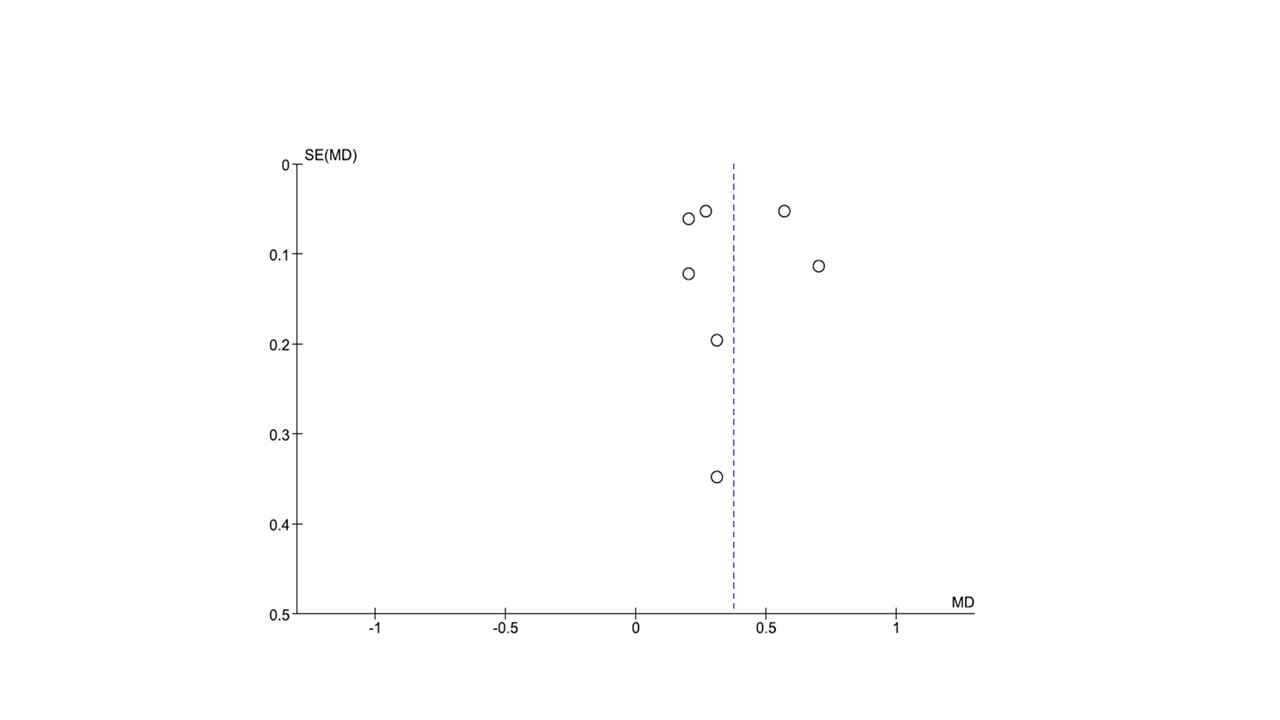


**Supplementary figure 1:** Funnel plot for publication bias analysis.

Supplement: Supplemental Digital Content [file medi-100-e28060-s001.doc]

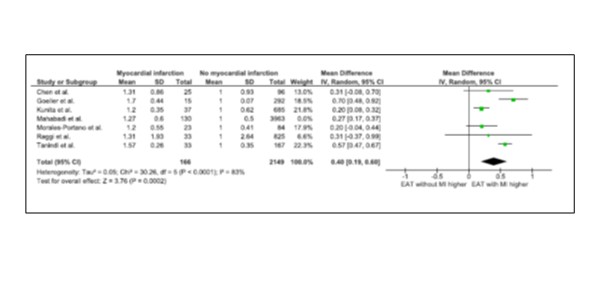


**Supplementary figure 2:** Forest plot without reference 12.

Supplement: Supplemental Digital Content [file medi-100-e28060-s002.doc]

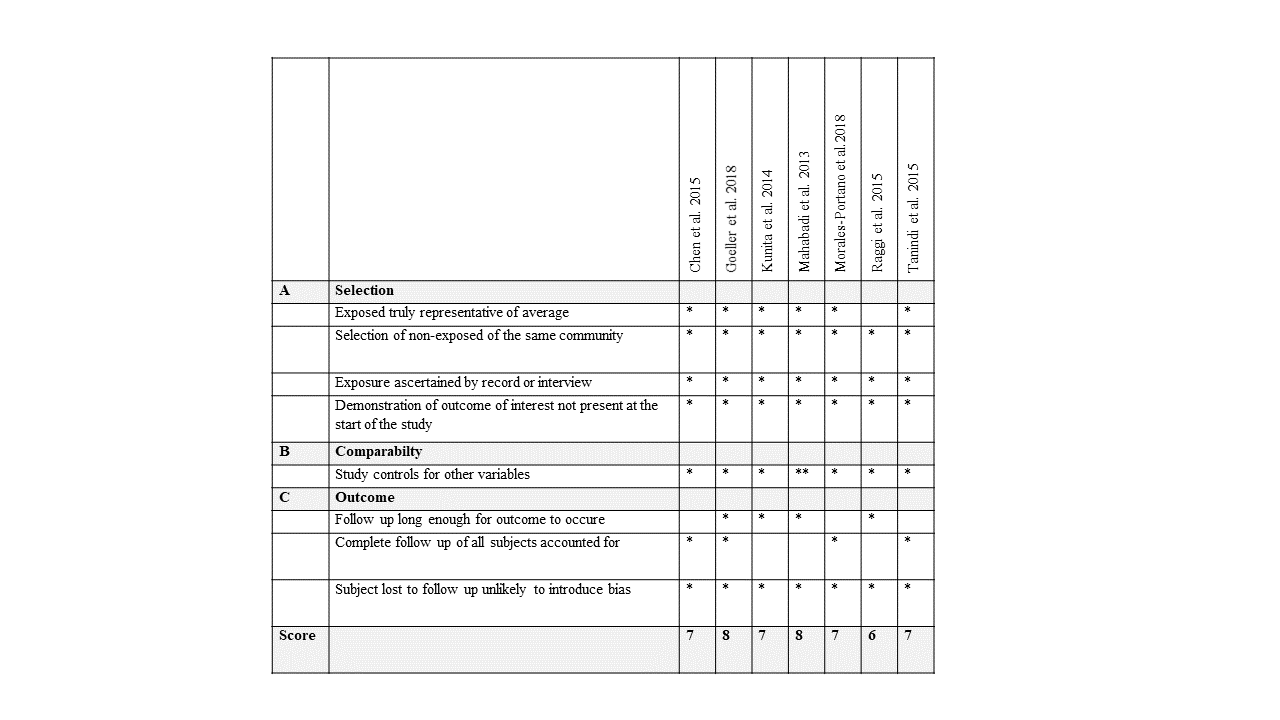


**Supplementary figure 3:** Newcastle Ottawa Scale

Supplement: Supplemental Digital Content [file medi-100-e28060-s003.doc]
